# Supplementary material for: Efficient method for isolation of high-quality RNA from Psidium guajava L. tissues
Source: PLoS One. 2021 Jul 26;16(7):e0255245. doi: 10.1371/journal.pone.0255245 (PMC8312961; doi:10.1371/journal.pone.0255245)
Supplement: S1 Fig — Table summarizing the quantification of the RNA samples in a NanoDrop spectrophotometer (Thermo Fischer, USA), as well as the ratios A260/A280 and A260/A230. Denaturing agarose gel (1%) electrophoresis of total extracted RNA (2 μL) stained with GelRed (Biotium, USA), original image. (DOCX) [file pone.0255245.s001.docx]

**
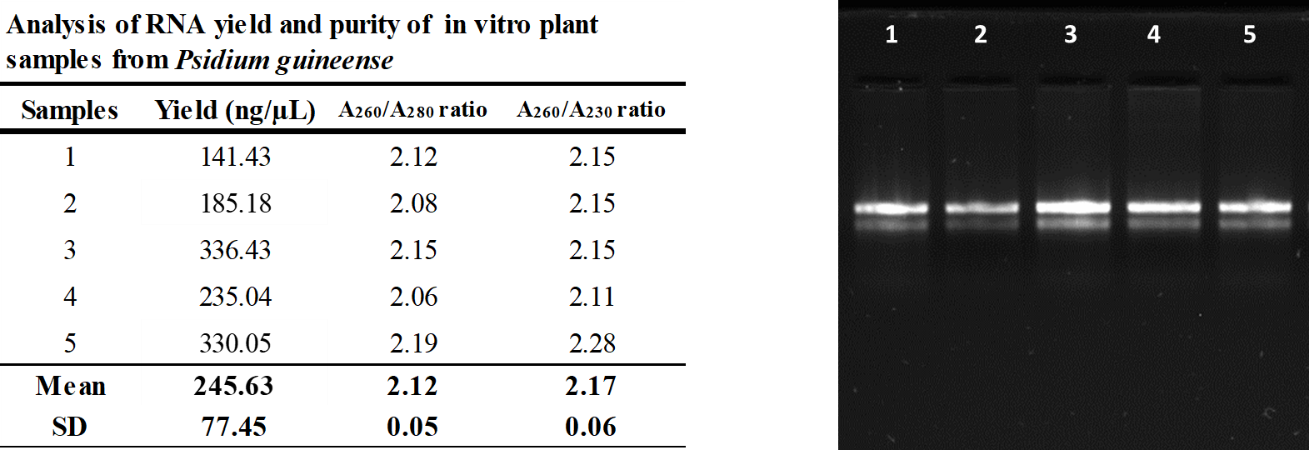
**

**S1 Fig. Analysis of RNA samples from *P. guineense*.** Table summarizing the quantification of the RNA samples in a NanoDrop spectrophotometer (Thermo Fischer, USA), as well as the ratios A_260_/A_280_ and A_260_/A_230_. Denaturing agarose gel (1%) electrophoresis of total extracted RNA (2 µL) stained with GelRed (Biotium, USA), original image.
