# Supplementary material for: Efficient method for isolation of high-quality RNA from Psidium guajava L. tissues
Source: PLoS One. 2021 Jul 26;16(7):e0255245. doi: 10.1371/journal.pone.0255245 (PMC8312961; doi:10.1371/journal.pone.0255245)
Supplement: S2 Fig — Five different tissues from P. guajava used to obtain the total RNA. Flower bud (FB); Immature leaf (IL); Young leaf (YL); Mature leaf (ML); and Root (R). (DOCX) [file pone.0255245.s002.docx]

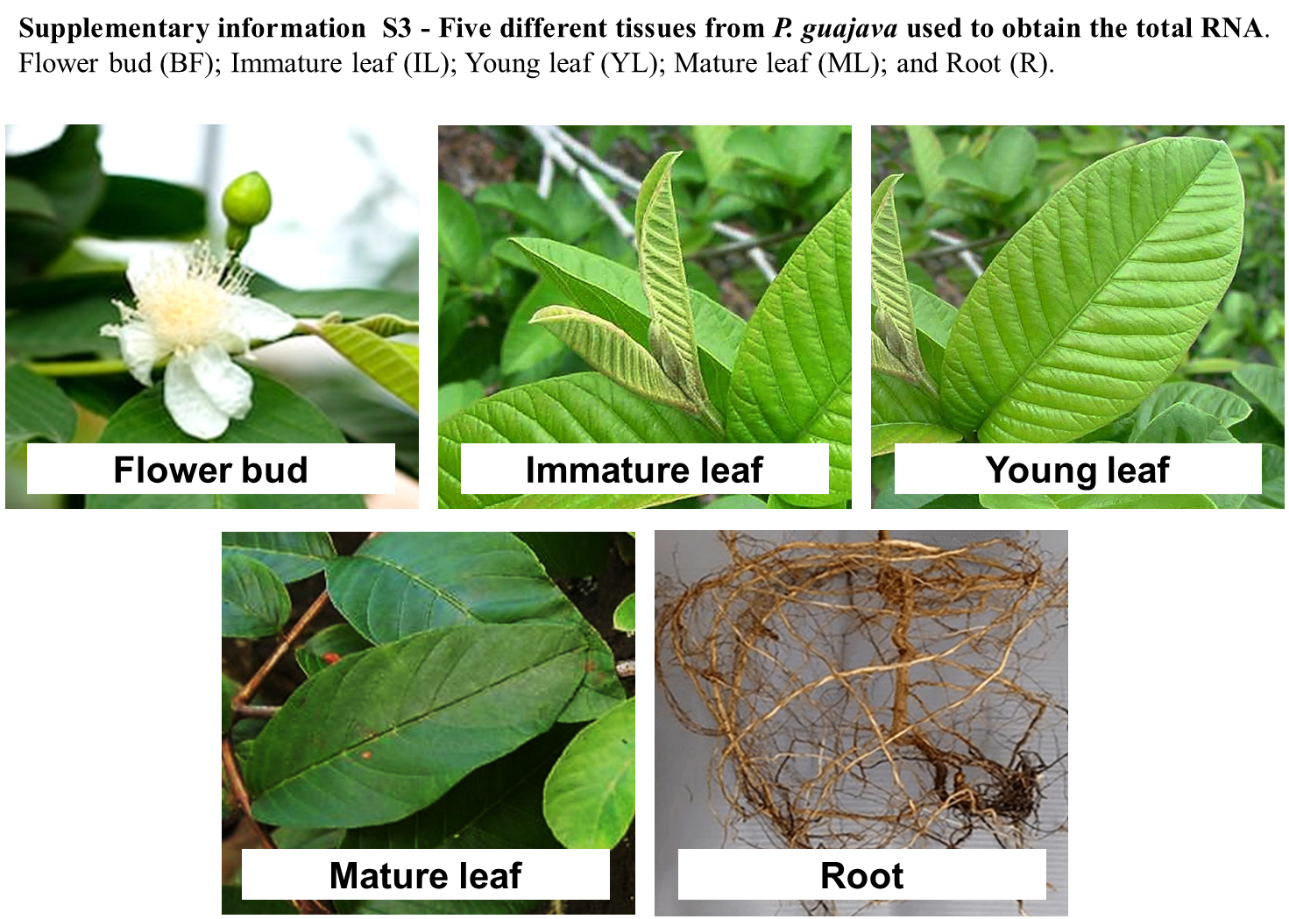


**S2 Fig. Images exemplification of tissues from *P. guajava* used in this study.** Five different tissues from *P. guajava* used to obtain the total RNA. Flower bud (FB); Immature leaf (IL); Young leaf (YL); Mature leaf (ML); and Root (R).
