# Supplementary material for: Efficient method for isolation of high-quality RNA from Psidium guajava L. tissues
Source: PLoS One. 2021 Jul 26;16(7):e0255245. doi: 10.1371/journal.pone.0255245 (PMC8312961; doi:10.1371/journal.pone.0255245)
Supplement: S3 Fig — For the primer efficiency analysis, standard curves were constructed with cDNA quantities of 50 ng (highest), 25 ng, 2.5 ng, 0.25 ng and 0.025 ng (lowest). The efficiency was 98%. (DOCX) [file pone.0255245.s003.docx]

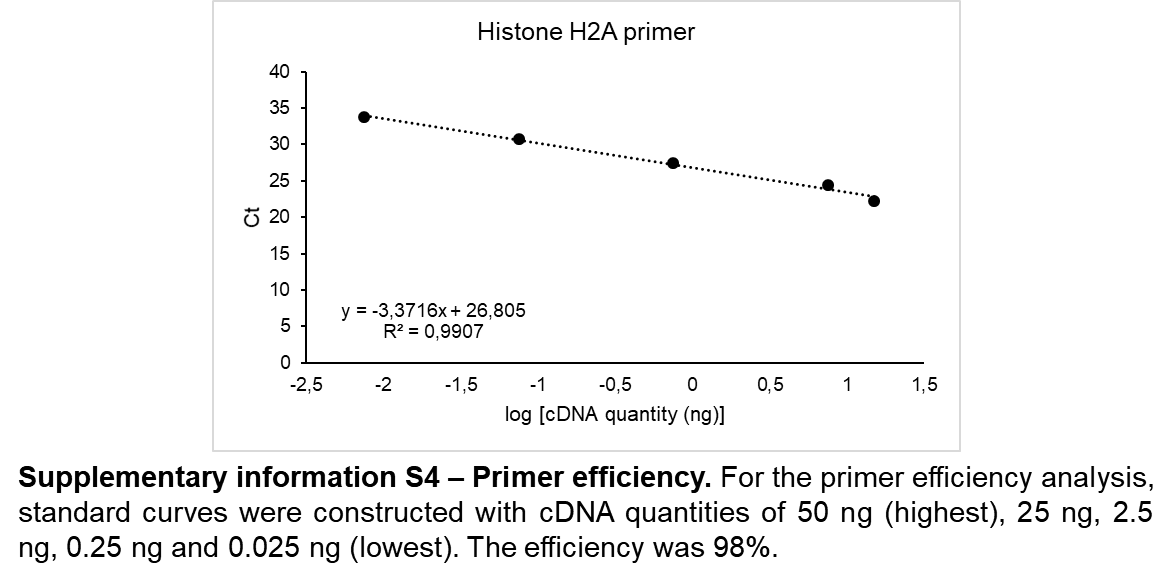


**S3 Fig. H2A primer efficiency.** For the primer efficiency analysis, standard curves were constructed with cDNA quantities of 50 ng (highest), 25 ng, 2.5 ng, 0.25 ng and 0.025 ng (lowest). The efficiency was 98%.
