# Supplementary material for: Efficient method for isolation of high-quality RNA from Psidium guajava L. tissues
Source: PLoS One. 2021 Jul 26;16(7):e0255245. doi: 10.1371/journal.pone.0255245 (PMC8312961; doi:10.1371/journal.pone.0255245)
Supplement: S4 Fig — 1-Ladder (faint); 2-Positive control (PureLink RNA Kit); 3- Sample1 (PureLink RNA Kit); 4- Sample2 (PureLink RNA Kit); 5- empty; 6- Positive control (RNeasy Plant Kit); 7- Sample1 (RNeasy Plant Kit); 8- Sample2 (RNeasy Plant Kit); 9- empty; 10- Positive control (CTAB1); 11- Sample1 (CTAB1); 12- Sample2 (CTAB1); 13- empty; 14- Positive control (CTAB2); 15- Sample1 (CTAB2); 16- Sample2 (CTAB2); 17- empty; 18- Positive control (TRIzol); 19- Sample1 (TRIzol); 20- Sample2 (TRIzol); 21- empty; 22- Positive control (Guanidine prorocol); 23- Sample1 (Guanidine prorocol); 24- Sample2 (Guanidine prorocol). Note that in Fig 1, we inverted the presentation of the results of the CTAB protocols with that of the commercial kits, to be more consistent with the presentation in the text. Therefore, we are presenting a cropped figure. (DOCX) [file pone.0255245.s004.docx]

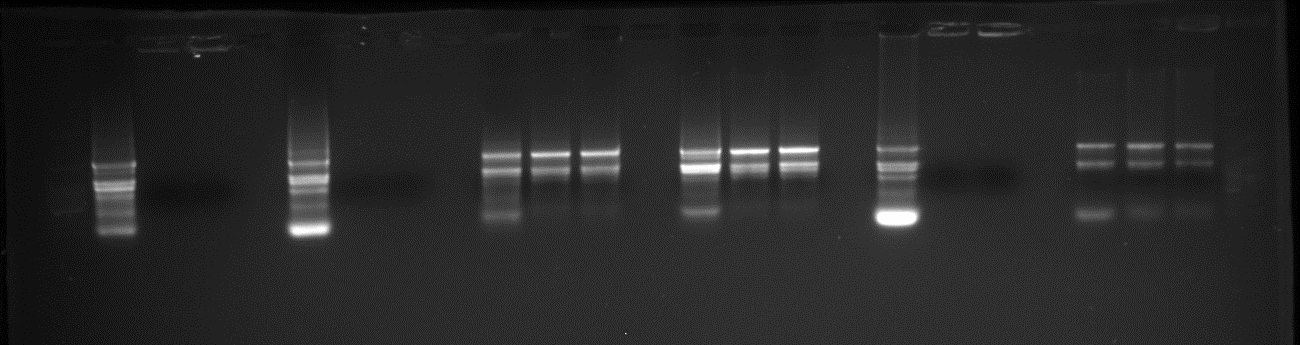


**S4 Fig. Original RNA agarose gel electrophoresis corresponding to Fig 1.** 1-Ladder (faint); 2-Positive control (PureLink RNA Kit); 3- Sample1 (PureLink RNA Kit); 4- Sample2 (PureLink RNA Kit); 5- empty; 6- Positive control (RNeasy Plant Kit); 7- Sample1 (RNeasy Plant Kit); 8- Sample2 (RNeasy Plant Kit); 9- empty; 10- Positive control (CTAB_1_); 11- Sample1 (CTAB_1_); 12- Sample2 (CTAB_1_); 13- empty; 14- Positive control (CTAB_2_); 15- Sample1 (CTAB_2_); 16- Sample2 (CTAB_2_); 17- empty; 18- Positive control (TRIzol); 19- Sample1 (TRIzol); 20- Sample2 (TRIzol); 21- empty; 22- Positive control (Guanidine prorocol); 23- Sample1 (Guanidine prorocol); 24- Sample2 (Guanidine prorocol). Note that in figure 1, we inverted the presentation of the results of the CTAB protocols with that of the commercial kits, to be more consistent with the presentation in the text. Therefore, we are presenting a cropped figure.
