# Supplementary material for: Efficient method for isolation of high-quality RNA from Psidium guajava L. tissues
Source: PLoS One. 2021 Jul 26;16(7):e0255245. doi: 10.1371/journal.pone.0255245 (PMC8312961; doi:10.1371/journal.pone.0255245)
Supplement: S6 Fig — Paluma Samples 1- Ladder (omitted); 2- Immature leaf1; 3- Immature leaf2; 4- Young leaf1; 5- Young leaf2; 6- Mature leaf1; 7- Mature leaf2; 8- Root1; 9- Root2; 10- empty. (DOCX) [file pone.0255245.s006.docx]

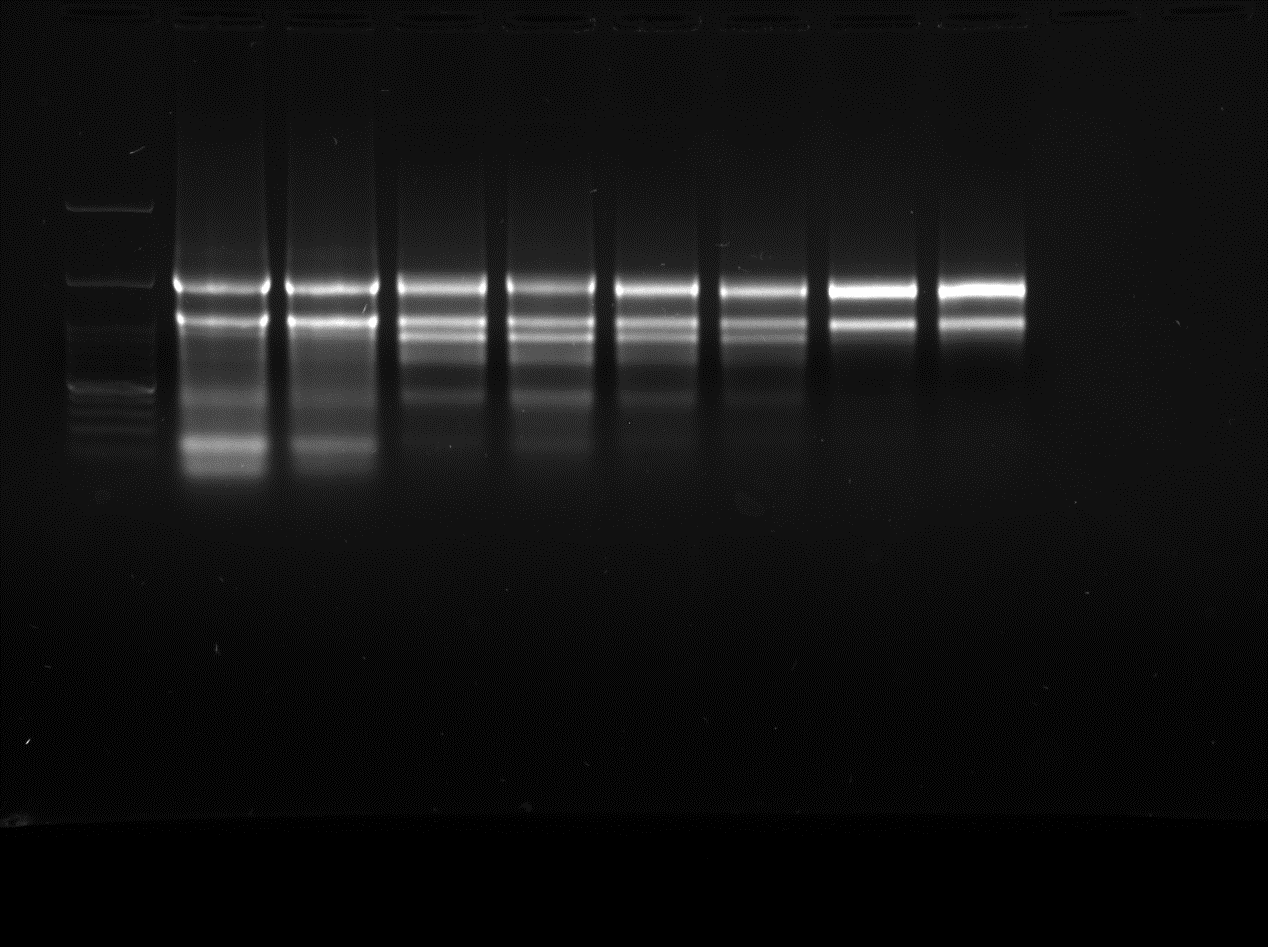


**S6 Fig. Original RNA agarose gel electrophoresis corresponding to Fig 2a.** Paluma Samples 1- Ladder (omitted); 2- Immature leaf1; 3- Immature leaf2; 4- Young leaf1; 5- Young leaf2; 6- Mature leaf1; 7- Mature leaf2; 8- Root1; 9- Root2; 10- empty.
