# Supplementary material for: Efficient method for isolation of high-quality RNA from Psidium guajava L. tissues
Source: PLoS One. 2021 Jul 26;16(7):e0255245. doi: 10.1371/journal.pone.0255245 (PMC8312961; doi:10.1371/journal.pone.0255245)
Supplement: S1 Table — Table summarizing the quantification of the RNA samples in a NanoDrop spectrophotometer, as well as the ratios A260/A280 and A260/A230. (DOCX) [file pone.0255245.s007.docx]

**
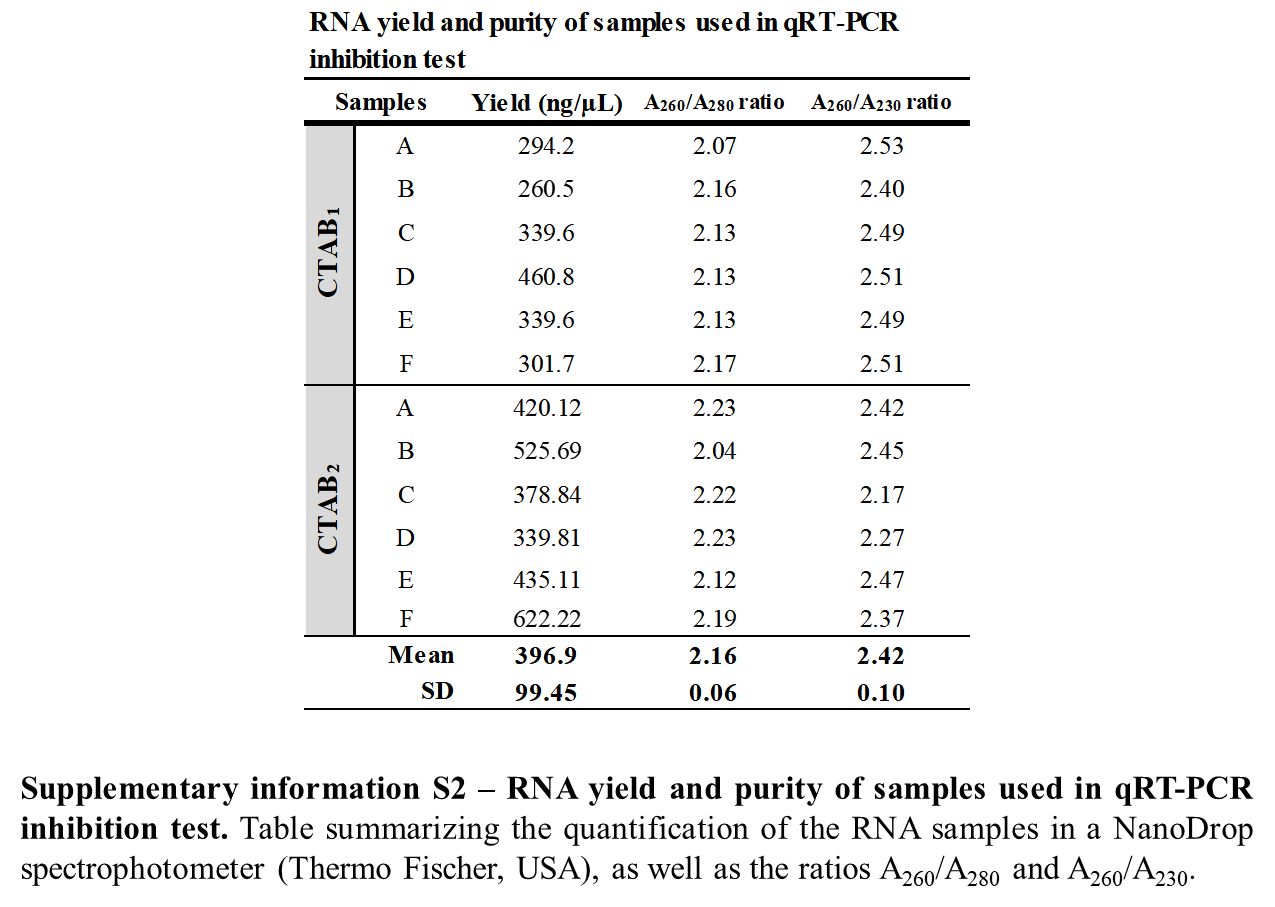
**

**S1 Table. RNA yield and purity of samples used in qRT-PCR inhibition test.** Table summarizing the quantification of the RNA samples in a NanoDrop spectrophotometer, as well as the ratios A_260_/A_280_ and A_260_/A_230_.
