# Supplementary material for: Efficient method for isolation of high-quality RNA from Psidium guajava L. tissues
Source: PLoS One. 2021 Jul 26;16(7):e0255245. doi: 10.1371/journal.pone.0255245 (PMC8312961; doi:10.1371/journal.pone.0255245)
Supplement: S1 File — (PDF) [file pone.0255245.s008.pdf]

RNA integrity was evaluated from the 28S and 18S rRNA bands in 1.0% (w/v) formaldehyde–agarose gel after electrophoresis, staining with 1:20,000 GelRed (Biotium, Fremont-CA – USA) and visualization with Gel Doc XR+ System (Bio-Rad, USA), using Image Lab software (auto scale). The method used to capture the image was the same for all agarose gel.

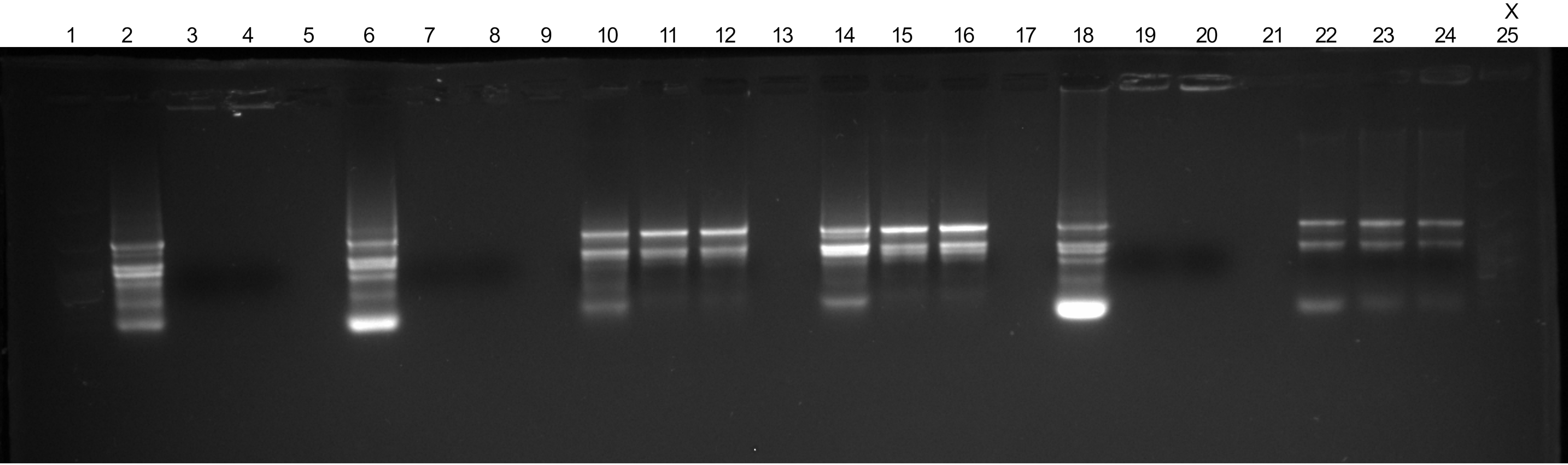

Raw data RNA agarose gel electrophoresis corresponding to Fig 1. 1- Molecular weight marker (faint); 2- Positive control (PureLink RNA Kit); 3- Sample1 (PureLink RNA Kit); 4- Sample2 (PureLink RNA Kit); 5- empty; 6- Positive control (RNeasy Plant Kit); 7- Sample1 (RNeasy Plant Kit); 8- Sample2 (RNeasy Plant Kit); 9- empty; 10- Positive control (CTAB1); 11- Sample1 (CTAB1); 12- Sample2 (CTAB1); 13- empty; 14- Positive control (CTAB2); 15- Sample1 (CTAB2); 16- Sample2 (CTAB2); 17- empty; 18- Positive control (TRIzol); 19- Sample1 (TRIzol); 20- Sample2 (TRIzol); 21- empty; 22- Positive control (Guanidine protocol); 23- Sample1 (Guanidine protocol); 24- Sample2 (Guanidine protocol); 25- Molecular weight marker (faint; omitted). Note that in figure 1, we inverted the presentation of the results of the CTAB protocols with that of the commercial kits, to be more consistent with the presentation in the text. Therefore, we are presenting a cropped figure.

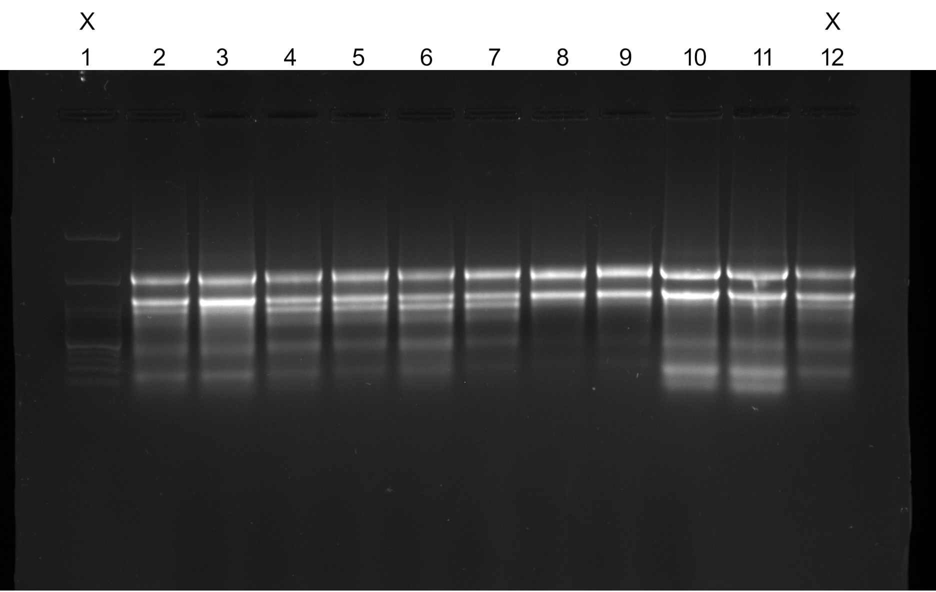

Raw data RNA agarose gel electrophoresis corresponding to Fig 2a. Cortibel Samples 1- Molecular weight marker (omitted); 2- Immature leaf1; 3- Immature leaf2; 4- Young leaf1; 5- Young leaf2; 6- Mature leaf1; 7- Mature leaf2; 8- Root1; 9- Root2; 10- Flower bud1; 11- Flower bud2; 12- Flower bud3 (omitted). Note that in figure 2A, we inverted the presentation of the results of the flower bud samples.

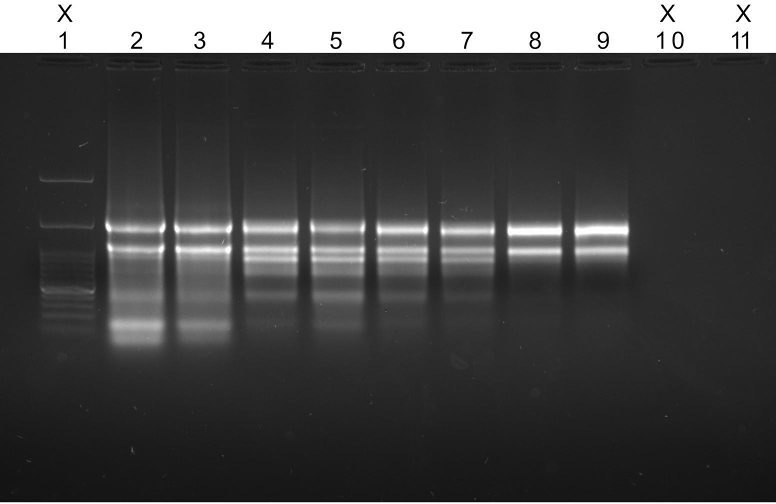

Raw data RNA agarose gel electrophoresis corresponding to Fig 2a. Paluma Samples 1- Molecular weight marker (omitted); 2- Immature leaf1; 3- Immature leaf2; 4- Young leaf1; 5- Young leaf2; 6- Mature leaf1; 7- Mature leaf2; 8- Root1; 9- Root2; 10- empty (omitted); 11- empty (omitted).

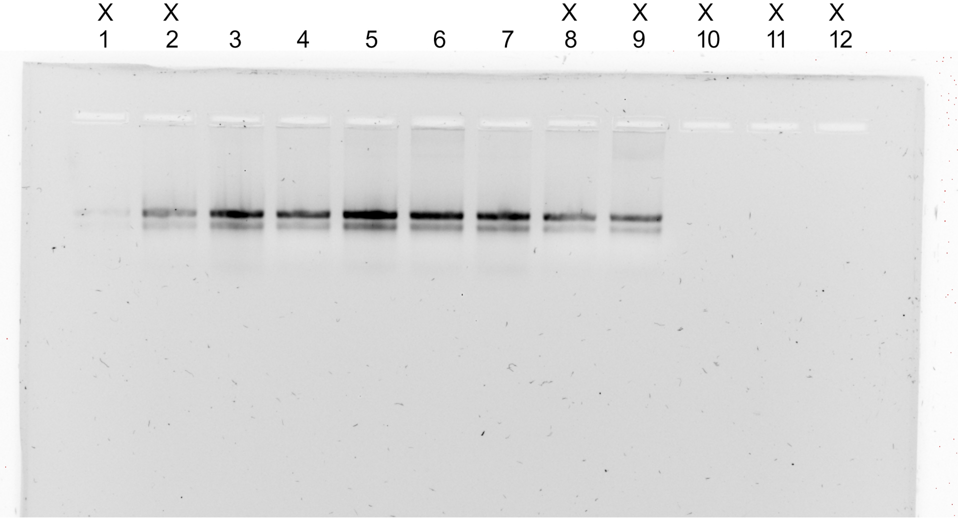

Raw data RNA agarose gel electrophoresis corresponding to Fig S1. Analysis of RNA samples from *P. guineense*. 1- *in vitro* sample (omitted); 2- *in vitro* sample (omitted); 3- leave sample1; 4- leave sample2; 5- leave sample3; 6- leave sample4; 7- leave sample5; 8- other species sample (omitted); 9- other species sample (omitted); 10- empty (omitted); 11- empty (omitted); 12- empty (omitted).
