# Supplementary material for: Efficient method for isolation of high-quality RNA from Psidium guajava L. tissues
Source: PLoS One. 2021 Jul 26;16(7):e0255245. doi: 10.1371/journal.pone.0255245 (PMC8312961; doi:10.1371/journal.pone.0255245)
Supplement: Attachment — Submitted filename: Certificates of English Editing.pdf [file pone.0255245.s011.pdf]

Editing by Professional Editors
(CNPJ 26.343.536/0001-01)

Date: July 8, 2021

CERTIFICATE OF ENGLISH EDITING

To whom it may concern,

This is to certify that the paper with the provisional title Efficient method for isolation of high-quality RNA from *Psidium guajava* L. tissues, to be submitted by Paola de Avelar Carpinetti, Vinícius Sartori Fioresi, Thais Ignez da Cruz, Francine Alves Nogueira de Almeida, Drielli Canal, Adésio Ferreira, Marcia Flores da Silva Ferreira, has been edited for language by M&M Academic Services. Neither the research content nor the author's intentions were altered in any way during the editing process.

M&M Academic Services guarantees the quality of English language in this paper, provided our editor's changes are accepted and further changes made to the paper are checked by our editor.

Matthew Ferreira, Ph.D., M.Sc., B.Sc.
President — M&M Academic Services

M&M ACADEMIC SERVICES
SÃO PAULO, SP, BRAZIL

academic.services.mm@gmail.com
https://academicservicesmm.wixsite.com/usa-brazil-1

TIKINET

CNPJ: 15.267.097/0001-70
R. Santanésia, 528 - 1° andar, Vila Pirajussara
CEP: 05580-055 | São Paulo – SP
(11) 2361-1808
www.tikinet.com.br

São Paulo, June 29, 2021

CERTIFICATE

TIKINET EDIÇÃO LTDA. – EPP, located at Rua Santanésia, 528, 1º andar, Cj. 11, São Paulo-SP, 05580-050, Brazil, has qualified professionals to perform the services of copyediting and translation of texts in foreign languages. We attest that the following hired services were performed in their totality.

Type of Service: (X) Copyediting / (   ) Translation

Language: English

Document: Efficient method for isolation of high-quality RNA from Psidium guajava L. tissues

....

Carlos Eduardo Chiba
Tikinet Edição Ltda. – EPP
CNPJ: 15.267.097/0001-70
