# Supplementary material for: Efficient method for isolation of high-quality RNA from Psidium guajava L. tissues
Source: PLoS One. 2021 Jul 26;16(7):e0255245. doi: 10.1371/journal.pone.0255245 (PMC8312961; doi:10.1371/journal.pone.0255245)
Supplement: Attachment — Submitted filename: response to reviewers.docx [file pone.0255245.s009.docx]

March 8, 2021.

Editorial Board

Dear Co-Editors-in-Chief, PLoS One

We would like to resubmit for publication as a Research Article in *Plos One* the manuscript “**Efficient Method for Isolation of High-quality RNA from *Psidium guajava* L. Tissues**” (MS-Ref No PONE-D-20-38857). Please find enclosed our response to reviewers’ comments, according to the advice of the Academic Editor.

**Response to reviewers’ comments**

*We are very grateful to the reviewers for their helpful and constructive comments on our manuscript. We hope the editor and the reviewers agree that we have adequately addressed the issues raised by the reviewers, and the manuscript is now suitable for publication in Plos One.*

**Reviewer #1:**

The manuscript titled "Efficient method for isolation of high-quality RNA from *Psidium guajava* L. tissues" investigates the efficiency of various RNA isolation methods from the intended plant. Although the manuscript is well composed, the following minor comments need to be addressed.

1-) The authors compared the RNA isolation protocols primarily based on the results from extracts of young leaves (Table 1), and then applied the best protocol to compare results from other tissues (Table 2, Figure 2). It would have been great if all the protocols were initially tested for each tissue since the level of accumulation of interfering metabolites varies between plant tissues/parts. The authors are advised to provide the outcomes if they performed such analysis.

**Answer:** *We agree with the referee that for a better comparison between the methods, all evaluated tissues should be used. However, since this was a pioneering study, we decided to first evaluate the effectiveness of these techniques on the most commonly used tissue source, the leaves. After identifying the CTAB2 method as the most effective technique, since it presented a better yield, good quality, low contamination with PCR interferences, as well as being a faster and easier method to execute than CTAB1, we evaluated other tissues. Having this as a foundation, our future studies will be able to specify and perfect the conditions of the technique used, including the tissue source.*

2-) The representations of sample numbers (1-5) and codes (A-F) in supporting information S1 and S2 are not clearly specified. Also, the results from each are poorly explained in the manuscript.

**Answer***: The supplementary information 1 - S1 presents the result of the extraction of five samples (numbered 1-5) of leaf tissue from plants of the species Psidium guineense. On the left is the table with the results of the NanoDrop analysis, which provided us with the yields (evaluated by absorbance at 260 nm) and quality of the samples (ratio 260/230 and 260/280). On the right is the image of the agarose gel evaluation of these five samples, which attests to the integrity of the RNA obtained. We decided to include this information in the manuscript because we consider it to be an indication that this protocol is also useful for the extraction of quality RNA from tissues of other species of the Psidium genus, which also have high concentrations of secondary metabolites and are, therefore, more difficult to nucleic acids.*

*Supplementary information 2 – S2 is related to figure 3. In this table we present the individual quantifications (obtained from the 260 nm length assessment) and their respective ratios (260/230 and 260/280) of all samples that were used in the RT-qPCR experiment (figure 3). In this experiment, six RNA samples (identified by the letters of A-F) obtained from the CTAB1 protocol and six samples obtained from the CTAB2 protocol were evaluated. We chose to include this data to present addition information that may help readers evaluate the experiment. However, by bringing information with a technical aspect, we saw no need to discuss it in the manuscript. If the reviewers or the editor believe that this information is not useful for the manuscript, we can remove it without harming the study.*

3-) Vendor details are missing for most of the chemicals and reagents used in the study, and a separate subsection is needed in the Methods part.

**Answer:** *We appreciate the recommendation and added the information in the manuscript, as highlighted in the text. We have chosen to use a single section “Materials and Methods” as the standard model suggested in the Plos One publication guide.*

4-) All trade mark signs (such as TM, ®,...) need to be removed from all reagent, chemical, and instrument names throughout the manuscript.

**Answer:** *We appreciate the recommendation and have corrected it in the manuscript, as highlighted in the text.*

5-) Table 1 and Table 2 are each presented twice, and the ones provided at the end of the manuscript should be removed.

**Answer:** *We apologize for the inconvenience, possibly this error occurred during the addition of the files to the submission platform, which generated a pdf file with duplicate tables.*

6-) Figure 3: The linear regression curves and associated data are invisible (in the pdf file) and hence, need to be replaced.

**Answer:** *We appreciate the recommendation and have corrected them in the figure 3.*

**Reviewer #2**

The authors describe in the manuscript the extraction of RNA from the Psidium guajava plant, where they analyzed 6 different methods, and determined that the CTAB method was the best. This work focused on finding the best methodology of previously reported protocols, making slight modifications, so I consider that the manuscript does not provide relevant information, only the authors found a protocol where they managed to extract RNA of good integrity and quality according to analysis in gels agarose and spectrophotometric measurements. However, when analyzing the samples by TapeStation Systems, the RIN values obtained were low, and despite performing RT-qPCR (with a single gene) I do not consider it to be sufficient analysis to confirm that it is a high-quality RNA (concentration and purity) and that it can be used for gene expression studies and other omic analysis as they describe in the document. Also, the document contains important errors. For this reason, I consider that the manuscript (in this version) should not be accepted by the journal.

**Answer:** *We appreciate the reviewer's considerations, but we believe that the publication of an RNA isolation method capable of extracting quality RNA from various tissues of P. guajava is of great use to the scientific community, since guava is a worldwide distributed plant, it has economic relevance and its use in molecular studies are increasing (Feng et al. 2020 – doi: 10.1111/pbi.13498). Our findings may assist research groups that intend to carry out molecular studies with this and other species of the genus, providing an easy-to-perform method that obtains RNA with a high yield and quality, avoiding unnecessary expenditure of time and financial resources. We also respectfully disagree that the RIN values are inadequate, since it is known that the calculation of these values is not accurate for plant RNA (due to additional sub-units of rRNA), as will be discussed in the comments below. Therefore, we would like you to evaluate our responses to your comments and reconsider the publication of the manuscript.*

Specific comments:

The authors mention the following (lines 39-40): “For the most efficient protocol, the average yield of RNA from guava leaf was 210.4 µg/g of tissue, and the A 260 /A 280 and A 260 /A 230 ratios were 2.1 and 2.2, respectively”. But of the two tables they present do not report that value, where did they get it from?

**Answer:** *In the table, the values presented refer to the concentration of RNA (ng/uL), calculated by NanoDrop. However, to address the amount of RNA obtained, it is necessary to present the values in microgram of RNA per gram of tissue, and for this calculation the concentration (table 1), the elution volume (described in the Material and Methods section), and the amount of starting tissue (described in the Material and Methods section) are used.*

Line 41. RT-PCR analysis, or they refer to analysis performed by real-time PCR (RT-qPCR).

**Answer:** *We appreciate the recommendation and have corrected it in the manuscript, as highlighted in the text.*

Line 97. Ref. [37] It is from Furlan et al., (2012), however in this article the methodology is not described, it refers to Salzman et al. (1999) and even mentions with modifications. For an article where the search for a good method is proposed, it is necessary to place the correct references.

**Answer:** *The method used by Furlan et al. was adapted from Salzman et al. Although this protocol is not fully detailed in the article by Furlan at al., we contacted the authors who cordially provided us with the detailed protocol used in that study, as described in the manuscript. For better understanding, we added this information in the manuscript (lines 442-444) and we also chose to add "modified Salzman el al. protocol”* *to avoid misunderstandings.*

Lines 98-99. "... in plant molecular biology studies". They must be more specific because molecular studies are several, and each one may have important considerations in the quality of the RNA, particularly for gene expression studies using RNA-seq where RIN values greater than 8.5 are required.

**Answer:** *We appreciate the comment, but as mentioned in other inquiries, the RIN values for plant samples are not accurate and may not reflect the quality of the RNA, as discussed in several manufacturer's application notes (Agilent). Among them, we provide a link to one that addresses why RIN is inaccurate for plant RNA. Link: https://www.agilent.com/cs/library/applications/5990-8850EN.pdf*

Figure 1. Place the name of the method and not the reference on the gel image. In table 1, if the name of the method is described which is confusing.
The Authors need to indicate how much was loaded on the agarose gels.
Figure 3 is unacceptable, nothing is observed. I can not express an opinion regarding the text that addresses figure 3 because it is not observed. The RNA concentrations obtained and that are mentioned in lines 141-143, do not coincide with what is reported in the tables. Why?. Even the units of concentration are different. However, the 260/230 ratio (1.63 ± 0.26) of the guanidine thiocyanate method reported does match. Why, despite having the same concentrations with the trizol and guanidine thiocyanate method, are there no bands in the agarose gel with the trizol extraction?

**Answer:** *We appreciate the recommendation about table 1, and corrected it in the manuscript.*

*We apologize for the inconvenience about figure 3, possibly this error occurred during the addition of the files to the submission platform, which generated a pdf file with duplicate tables.*

*Regarding the question concerning the values presented in the results being different from those shown in the tables (as explained in the previous comment), these values do not refer to the same information, for this reason they are different. The table shows the RNA concentration data (ng of RNA per uL), while in this part of the text (Result Section) we present the normalized total RNA quantity (microgram of RNA obtained per gram of tissue).*

*As to why it is not possible to see bands on the agarose gel for samples of the TRIzol method, despite having the same concentration as the Salzman et al. method, as we discussed in the manuscript (lines 148-154), this possibly occured because some contaminants (e.g. guanidine and phenol) that absorbs at a wavelength of 260 nm (even used for nucleic acid quantification) can distort that quantification. This reasoning is evidenced by the low 260/230 ratio, indicating an unexpected absorption for nucleic acids at a wavelength of 230 nm (but expected absorption for phenol and guanidine).*

In the supplementary figures, DNA remains are shown, the authors had to add a figure after treating the RNA with DNase I. Also, in the supplementary figures, they should add numbers or letters to identify the lines of the gels, as well as they are not clear. All gel images should have better contrast.

**Answer:** *Regarding the treatment with DNase I, it was only performed for the samples evaluated by TapeStation because this same sample set was used for RNA-seq experiment and this pretreatment with DNase I is a standard procedure (defined by the service provider sequencing). For this reason, we opted to add the original gels to the supplementary information, without any adjustments, and with maximum exposure (limit to avoid band saturation). The gels presented in figures 1 and 2, were obtained automatically by the Image Lab software (Bio-Rad), according to the best exposure and saturation times.*

The authors in the conclusion mention having obtained high quality RNA, however the agarose gels show traces of DNA and the analysis carried out in TapeStation Systems show very low RIN values, except for root, so I do not consider the CTAB method be effective for other molecular studies, such as RNA-seq to determine gene expression.

**Answer:** *Unfortunately, as informed by the manufacturer (Agilent), there is an important difficulty in assessing the RIN of chlorophyllated samples. First, plant rRNA gives different peaks than mammalian rRNA (standard for RIN calculations). Second, the choloroplast rRNA peaks are recognized as degradation products in the analysis and, hence, give lower RIN values. For this reason, the root tissue has better RIN than the others; however, it does not mean that the leaf samples are degradaded. According to Agilent’s protocol mentioned before, the range for RIN values in plants were between 6.4 and 8.4 in that report. Therefore, beyond the main findings of our study, we also will be contributing to the botanical literature in this sense. For this reason, the values obtained in these studies suggest a high quality of the samples, since if there were significant degradation, the RIN obtained should be even lower (lines 218-227).*

*As previously mentioned, part of these samples were used for transcriptome studies (RNA-seq) and all the libraries obtained were of high quality, as well as the generated sequences had the highest scores, demonstrating the efficiency of the method for this application (data not shown).*

Why did they use soybean as a positive control?. They do not justify it.

**Answer:** *We used soybean tissue as a positive control due to the ease of obtaining nucleic acids. In addition, it is a well-studied plant and for most of the methods evaluated in this study, RNA was extracted with high efficiency, including for commercial kits. We included this information in the manuscript, as highlighted in the text (lines 320-323).*

Line 316, need to add reference number.

**Answer:** *We appreciate the recommendation and corrected it in the manuscript, as highlighted in the text.*

Line 336, need to add reference number

**Answer:** *We appreciate the recommendation and corrected it in the manuscript, as highlighted in the text.*

Lines 411-412, need to add reference number

**Answer:** *We appreciate the recommendation and corrected it in the manuscript, as highlighted in the text.*

Page 21, lines 460-462. The Authors need to include the plant DNA source used in the design of said primers.

**Answer:** *We appreciate the recommendation and corrected it in the manuscript, as highlighted in the text (lines 497-500). The primer pair was designed from a gene sequence obtained after analyzing the orthologous genes of the H2A gene of Eugenia uniflora (species of the Myrtaceae family) using a draft of the P. guajava genome obtained by our laboratory (unpublished data).*

All protocols were described in the methodology as previously reported and the only modification was the amount of plant material, I think the whole description is not necessary.

**Answer:** *We chose to detail the protocols used because, as initially informed in the text, we made minor adjustments. We also chose to describe the entire procedure, from tissue disruption to adding the buffer until solubilizing the RNA, because we believe that these changes represent important steps that are not equally detailed in the original articles. Additionally, we added in the manuscript the steps that made these minor adjustments.*

Reviewer #3:

1-) Table 1 and 2 has been given twice in the manuscript and needs to be corrected.

**Answer:** *We apologize for the inconvenience. We adjusted the .doc file and believe that this duplication has been resolved.*

2-) Zymo research has given similar protocol for RNA isolation "How To Extract RNA From Polysaccharide-Rich Samples" this needs to be discussed in the manuscript particularly, how present protocol is better than Zymo Research suggested method.

**Answer:** *We appreciate the reviewer's suggestion, however, we chose these six protocols for extracting RNA from plants because they were the most recurrent in our bibliographic research and their commercial kits are widely used worldwide.*

3-) Direct-zol™ RNA Miniprep Kit from Zymo Research needs to be used for comparison.

**Answer:** *We are grateful for the reviewer's suggestion, however, among the various RNA extraction protocols available and commercial kits, we had to choose the most used ones. We believe that this protocol from Zymo Research could be useful for future analyses, but it would be impracticable for us to repeat all experiments to include this protocol at this moment. However, we would like to point out that among the protocols evaluated, our study was able to demonstrate that the CTAB2 protocol is a method capable of extracting high-quality, high-yield RNA for various tissues of P. guajava, fulfilling the purpose of the manuscript.*

**Reviewer #4:**

The authors have successfully isolated good quality RNA from P. guajava using CTAB methods, this will be useful to others whose work require high-quality RNA from the plant. However, the manuscript is not recommended for consideration for publication in its current form. Improvement is needed before it can be re-considered.
Please check for grammatical errors and typographical errors (throughout the whole manuscript) thoroughly to improve the manuscript. The writing style, sentence structure and language throughout requires improvement. It is also recommended to state the cultivars/genotypes in the title.

**Answer:** *We appreciate the comments and we have sent the manuscript for editing by a native speaker to correct grammar and syntax errors. We believe that after this review the manuscript is presented in an intelligible fashion and written in standardized English.*

Specific comment:

Line30: ‘Furthermore, there is still no tested….’ (Revise the word ‘tested’ in this sentence because RNA has been isolated from P. guajava (Mittal et al., 2020) although using different methods, and one published method was also used in the current study (Furlan et al., 2012)).

**Answer:** *We appreciate the recommendation and corrected it in the manuscript, as highlighted in the text.*

Line 41: Change ‘RT-PCR’ to ‘qPCR’ or ‘RT-qPCR’

**Answer:** *We appreciate the recommendation and corrected it in the manuscript, as highlighted in the text.*

Line 116: CTAB1 and CTAB2

**Answer:** *We appreciate the recommendation and corrected it in the manuscript, as highlighted in the text.*

Line 116 & 129: change ‘guanidine thiocyanate’ to ‘Furlan et al method’ (To avoid confusion as TRIzol, RNeasy and Purelink are also guanidine thiocyanate based).

**Answer:** *We appreciate the recommendation and corrected it in the manuscript, as highlighted in the text.*

Line 116: ‘….28S and 18S rRNA…’ (please check again)

**Answer:** *We apologize for the mistake and corrected it in the manuscript, as highlighted in the text.*

Line 120: ‘…no RNA band….’ (insert the word ‘RNA’)

**Answer:** *We appreciate the recommendation and corrected it in the manuscript, as highlighted in the text.*

Line 146: ‘…reflection of contaminants…’ (give examples of contaminants)

**Answer:** *We appreciate the recommendation and added this information in the manuscript, as highlighted in the text.*

Line 186: ‘It is known that the composition ….’ (what composition is the author referring to?)

**Answer:** *We appreciate the recommendation and corrected it in the manuscript, as highlighted in the text.*

Line 213: ‘The best results were obtained for immature leaf and root tissues…..’ (for which genotype is this?)

**Answer:** *TapeStation analysis was performed only with samples from the cultivar, Cortibel. We added this information in the figure 2.*

Line 215: RIN for all RNA samples isolated from the five tissue types for both genotypes should be provided. The RIN is important to verify the quality of the RNA sample, which reflects the title of this manuscript.

**Answer:** *We agree with the reviewer, however, due to financial constraints we were unable to perform the analysis by TapeStation for all samples. We performed this analysis only for* *ten samples that were used to obtain the transcriptome (a parallel study), for which we hired a specialized service for sequencing of these samples, which included the analysis by TapeStation. For better understanding, we modified the figure 2 and presented the RIN values for all samples. In addition, we would like to point out that RIN is not efficiently calculated for plant leaf tissues, as indicated by Agilent. Since plant rRNAs have different peaks than mammalian rRNAs, such as the peaks referring to chloroplast rRNA these divergent peaks are recognized as degradation. Thus, the RIN value obtained for plant tissues will necessarily be lower, even with equivalent integrity.*

Line 278: ‘…. showing the advantage of lower cost and time investment. ‘ (lower time investment compared to?)

**Answer:** *We appreciate the recommendation and corrected it in the manuscript, as highlighted in the text.*

Line 296: The amount of tissue used for each extraction should be indicated specifically. Starting material of 80-100mg of tissue can make a lot of different in RNA yield and concentration obtained. Was the same amount of tissue for the same sample used the same across methods?

**Answer:** *We appreciate the reviewer's attention and rewrote the text to make the quantities clearer. In the comparison tests, we used leaf discs (~ 100mg), so the variation between samples was insignificant, less than 3% by weight. Regarding the test with the other tissues, due to previous experiments, we chose to reduce the amount of flower bud tissue (~80 mg) and increase the amount of root tissue (~200mg), to obtain better results.*

Line 317: State all the modifications made specifically. Any other modification made other than the reduction of starting material?

**Answer:** *We chose to detail our procedure and not report each change, since we believe that it would make the text more truncated and confusing (lines 339-340 and 363-364). However, as suggested by the reviewer, we describe in the manuscript the steps in which we made such changes.* *In the original protocol for CTAB1, the authors detail the order of addition of the reagents, for example the lysis buffer is added to the crushed tissue that is inside the tube, and to minimize losses, we chose to add the buffer in the mortar (as detailed in manuscript). Another modification (in addition to the starting material) was the two-step spin speed, which was originally 30,000 but we used 25,000 xg (due to the limitation of our centrifuge). In the CTAB2 protocol, the modifications in addition to the starting material were the concentration of LiCl (originally it was 4M but we used 5M), and the precipitation time which in the original article was suggested to be overnight but, to speed up the extraction (after previous tests), we opted for 4h time in a freezer at -20 °C. These modifications improved the purity of the RNA obtained and significantly reduced the time for extraction.*

Line 326& 361: ‘….and a new centrifugation was….’ (find more suitable words to replace ‘a new’)

**Answer:** *We appreciate the recommendation and corrected it in the manuscript, as highlighted in the text.*

Line 335: Is the extraction buffer for CTAB2 the same as the one used in CTAB1?

**Answer:** *Yes, the differences between these two protocols are in terms of procedures.*

Line 339: ‘….the mixture was stirred…’ (What apparatus was used to stir the mixture?)

**Answer:** *We appreciate the recommendation and corrected it in the manuscript, as highlighted in the text (line 365). “…the mixture was stirred using a pestle until becoming homogeneous.”*

Line345: Was the supernatant transferred to a new tube prior to the addition of 5M LICI?

**Answer:** *We appreciate the recommendation and corrected it in the manuscript, as highlighted in the text (lines 370-371). “The supernatant was carefully collected and transferred to a new 1.5 mL microtube. Next, 0.5 volume of 5 M LiCl was added to the supernatant…”*

Line 407: suggest to change the title for this subtopic from ‘Guanidine Thiocyanate method’ to ‘Furlan et al method’ (TRIzol, RNeasy and Purelink are also guanidine thiocyanate-based)

**Answer:** *We appreciate the recommendation and corrected it in the manuscript, as highlighted in the text.*

Line 422: ‘The RNA was then precipitated by the addition of 2 volumes of cold absolute ethanol and 0.1 volume of 5 M NaCl’ (State the duration and temperature used)

**Answer:** *We appreciate the recommendation and corrected it in the manuscript, as highlighted in the text.*

Line 466: Both CTAB1 and CTAB2?

**Answer:** *Yes. To make this information clearer, we added the description "CTAB1 and CTAB2", as highlighted in the text.*

Line 235 & 468: provide information on the concentration or amount of the RNA sample, in addition to the volume.

**Answer:** *This information (for each sample used) is already available in the supplementary information table S2.*

Figure 1: change the label ‘Salzman et al.’ to ‘Furlan et al’. Author tested the protocol of Furlan et al in this study (refer to statement in Line 97 and 167, and methodology). Similar change should be made in the text (example: line 116)

**Answer:** *We appreciate the suggestion, however, as suggested by reviewer #2, we standardized for Salzman et al. due to the fact that Furlan et al. had not detailed the protocol in the article. We describe this situation in the Material and Methods section (lines 418-4*
