# Supplementary material for: Efficient method for isolation of high-quality RNA from Psidium guajava L. tissues
Source: PLoS One. 2021 Jul 26;16(7):e0255245. doi: 10.1371/journal.pone.0255245 (PMC8312961; doi:10.1371/journal.pone.0255245)
Supplement: Attachment — Submitted filename: Response to Reviewers.docx [file pone.0255245.s010.docx]

*May 17, 2021.*

*Editorial Board*

*Dear Co-Editors-in-Chief,*

*We are very grateful to the reviewers for their helpful and constructive comments on our manuscript. We hope the Academic Editor and the reviewers agree that we have adequately addressed the issues raised by the reviewers, and the manuscript is now suitable for publication in Plos One.*

**Academic Editor’s comment:**

**1- Full vendor details should include company, city (state), and country. Please check and amend throughout the text.**

**Answer:** *We appreciate the recommendation and we have corrected it in the manuscript, as highlighted in the text.*

**2- Please don't use "We, our". Use impersonal phrasing throughout the text.**

**Answer:** *We appreciate the recommendation and we have added the information in the manuscript, as highlighted in the text.*

**3- Fig. 3 is not good, redo it in color.**

**Answer:** *We appreciate the recommendation and we have corrected it.*

**4- Before conclusion please add these two section:**

**a- Comparisons with other methods: You should compare your finding with other techniques to prove the applicability, if any. Summarize the findings in Table.**

**Answer:** *We appreciate the editor's suggestion; however, we believe that this information would be most appropriate if placed immediately after the results of comparison of protocols. Thus, we have included in this revised version the new suggested table (table 2 - line 163).*

**b- "Study strength and limitations". In this section, you have to discuss the strength and limitations of this study.**

**Answer:** *We appreciate the editor's suggestion. We have included this section in the revised version (lines 321-340).*

**5- Proofread the text for grammar and syntax errors.**

**Answer:** *We sent the manuscript back for editing by a native speaker to correct grammar and syntax errors. We believe that after this review the manuscript will be presented in an intelligible form and written in standardized English.*

**Journal Requirements:**

**When submitting your revision, we need you to address these additional requirements.**

1. **Please ensure that your manuscript meets PLOS ONE's style requirements, including those for file naming. The PLOS ONE style templates can be found at**

**Answer:** *We have corrected it in the revised manuscript.*

**PLOS ONE now requires that authors provide the original uncropped and unadjusted images underlying all blot or gel results reported in a submission’s figures or Supporting Information files. When you submit your revised manuscript, please ensure that your figures adhere fully to these guidelines and provide the original underlying images for all blot or gel data reported in your submission.**

**In your cover letter, please note whether your blot/gel image data are in Supporting Information or posted at a public data repository, provide the repository URL if relevant, and provide specific details as to which raw blot/gel images, if any, are not available. Email us at** [**plosone@plos.org**](mailto:plosone@plos.org) **if you have any questions.**

**Answer:** *The gel images data are in Supporting Information.*

3. PLOS requires an ORCID iD for the corresponding author in Editorial Manager on papers submitted after December 6th, 2016. Please ensure that you have an ORCID iD and that it is validated in Editorial Manager.

**Answer:** *The ORCID iD of the corresponding author is validated.*

**Reviewer #1’s comment:**

The authors have addressed most of the issues raised during the first review process. In order to clearly evaluate the efficiency of the different extraction protocols, however, statistical analysis should be conducted on the data in Table 1 and Table 2 (i.e the authors should evaluate and indicate if there is any significant difference in yield in relation to variation in extraction protocol, tissue type, and genotype). The result and discussion parts should also describe these statistical analysis results.

**Answer:** *We appreciate and agree with the reviewer's comments, and modified tables 1 and 2, including statistical analysis, as well as adding this information in the discussion (lines 155-162 and 223-231).*

**Reviewer #2’s comment:**

The manuscript is technically sound and all the suggestions given by the reviewers has been addressed and necessary corrections has been done to improve the manuscript. The data presented in the manuscript is sufficient and well analyzed as per the objective of the experiments. All the data are available in the manuscript and its supplementary part. There is no additional comments to authors.

**Answer:** *We appreciate the comments.*

**Reviewer #3’s comment:**

The authors responded adequately to the questions posed and have improved the manuscript, for which I consider it convenient for its publication in this journal.

Minor comments

Line 116, 129, 142, 146. Since the authors added this reference in the Results and Discussion section, they must add the reference number.

**Answer:** *We appreciate the recommendation and we have added the information in the manuscript.*

Line 235-237. True, the authors demonstrated to obtain high-quality RNA, however, in this section it could be convenient (if the authors consider) to add that this RNA obtained may still have limitations for other molecular studies such as gene expression analysis by RNA-seq (among other) , due to the RIN values obtained, and for the reasons mentioned in previous paragraphs. Examples of Ref. (Doi: 10.1186 / 1471-2199-7-3 and <https://doi.org/10.1016/j.mimet.2020.105855>).

**Answer:** *We appreciate the recommendation and we have added the information in the manuscript (lines 244-255).*
